# Genome-wide Diversity and Association Mapping for Capsaicinoids and Fruit Weight in *Capsicum annuum* L

**DOI:** 10.1038/srep38081

**Published:** 2016-11-30

**Authors:** Padma Nimmakayala, Venkata L. Abburi, Thangasamy Saminathan, Suresh B. Alaparthi, Aldo Almeida, Brittany Davenport, Marjan Nadimi, Joshua Davidson, Krittika Tonapi, Lav Yadav, Sridhar Malkaram, Gopinath Vajja, Gerald Hankins, Robert Harris, Minkyu Park, Doil Choi, John Stommel, Umesh K. Reddy

**Affiliations:** 1 Gus R. Douglass Institute and Department of Biology, West Virginia State University, Institute, WV-25112, USA; 2 Department of Plant Science, Plant Genomics and Breeding Institute, College of Agriculture and Life Sciences, Seoul National University, Seoul 151-321, Republic of Korea; 3 Genetic Improvement of Fruits and Vegetables Laboratory (USDA, ARS), Beltsville, MD-20705, USA.

## Abstract

Accumulated capsaicinoid content and increased fruit size are traits resulting from *Capsicum annuum* domestication. In this study, we used a diverse collection of *C. annuum* to generate 66,960 SNPs using genotyping by sequencing. The study identified 1189 haplotypes containing 3413 SNPs. Length of individual linkage disequilibrium (LD) blocks varied along chromosomes, with regions of high and low LD interspersed with an average LD of 139 kb. Principal component analysis (PCA), Bayesian model based population structure analysis and an Euclidean tree built based on identity by state (IBS) indices revealed that the clustering pattern of diverse accessions are in agreement with capsaicin content (CA) and fruit weight (FW) classifications indicating the importance of these traits in shaping modern pepper genome. PCA and IBS were used in a mixed linear model of capsaicin and dihydrocapsaicin content and fruit weight to reduce spurious associations because of confounding effects of subpopulations in genome-wide association study (GWAS). Our GWAS results showed SNPs in Ankyrin-like protein, IKI3 family protein, ABC transporter G family and pentatricopeptide repeat protein are the major markers for capsaicinoids and of 16 SNPs strongly associated with FW in both years of the study, 7 are located in known fruit weight controlling genes.

The pepper genus *Capsicum* originated in Bolivia and consists of 25 to 30 species; five are domesticated: *C. annuum, C. baccatum, C. chinense, C. frutescens,* and *C. pubescens*^1^,^2^. *C. annuum* is the most popular and was first introduced from the West Indies to Europe in March 1493, with the first travels of Christopher Columbus^3^,^4^. Trade routes between Europe, Middle-East and Asia promoted additional introductions and reciprocal exchanges, so multiple introductions were rapidly cultivated in most tropical, Mediterranean and temperate regions of the world. In these secondary diversification centers, thousands of landraces have been selected for 4 to 5 centuries by growers to fit new environments and local consumption habits and trade, which has resulted in wide phenotypic diversity of pepper cultivars^5^,^6^,^7^.

For many crop species, identification and utilization of single nucleotide polymorphisms (SNPs) have become economical because of the availability and abundance of various high-throughput technologies. Recently, three whole-genome sequence (WGS) drafts of hot pepper were released for public use, which opened up unique opportunities for pepper research. The WGS for *C. annuum* cv. CM334 is 650.2 Gb (186.6× genome coverage)^8^. WGS for *Zunla-1 (C. annuum* L.) and its wild progenitor *Chiltepin (C. annuum* var. *glabriusculum*) were made available by Qin *et al*.^9^. An examination of population structure, diversity at the molecular level, linkage disequilibrium (LD) distribution across various chromosomes and quantitative trait loci (QTL) estimated by model-based association mapping would provide insights into the evolution of various traits among *C. annuum* cultivars^7^. LD distribution information across the *C. annuum* genome will help group SNPs into haplotypes and, their use in genome analysis can lead to understanding the consequences of selection and breeding histories across the collections of *C. annuum* L.

Genome-wide association study (GWAS) with SNPs generated by genotyping by sequencing (GBS) has been widely used in all major crops including maize, rice, barley, tomato, wheat, sorghum, soybean, watermelon and several other important plant species^10^,^11^,^12^,^13^,^14^ and found effective for mining new genes; however, the population structure must be resolved accurately to reduce spurious associations because of confounding effects of subpopulations. The current research aims to identify genomic segments linked to various fruit traits and capsaicin accumulation in diverse collections of *C. annuum*. Despite several QTL studies of pepper^15^,^16^,^17^,^18^,^19^,^20^, the current study is unique in that it utilizes WGS drafts for anchoring SNPs and systematic GWAS pipelines to identify SNP markers for various fruit-related traits with special reference to capsaicinoid content and fruit weight (FW). It aims to identify genome-wide effects on capsaicinoid content and fruit weight (FW) in *C. annuum* populations by using SNPs identified by GBS.

## Results

### SNP development

A total of 250,493,661 reads were available for analysis after quality trimming and 56.0% of these were aligned to unique positions and 24% to multiple positions on the physical map. Accession-wise reads are presented in Table S2. A total of 66,960 SNPs were identified from the reads obtained for the 94 diverse *C. annuum* accessions studied; 7678, 7751, 8201, 6285, 4724, 6087, 5230, 3873, 3826, 4279, 3768 and 5268 SNPs were mapped to the WGS draft (Kim *et al*.^8^) and located on chromosomes 1, 2, 3, 4, 5, 6, 7, 8, 9, 10, 11 and 12, respectively. When screened for minor allele frequency (MAF) of 0.05% and call rate > 95%, total SNPs were reduced to 7,331 with 839, 683, 1007, 459, 566, 715, 464, 423, 479, 641, 543 and 512 on chromosomes 1 to 12, respectively. In addition, we identified 2,521 SNPs (containing MAF 0.05) located in various exons. SNPs in exons of various genes were 288, 295, 346, 131, 183, 231, 162, 153, 154, 218, 164 and 196 on chromosomes 1, 2, 3, 4, 5, 6, 7, 8, 9, 10, 11 and 12, respectively. We noted presence of one SNP at every 40.7 kb across the genome, with average gap size of 26.08 kb, and one SNP at every 78.8 kb in the coding regions. After excluding for MAF present at 0.05%, we noted one SNP at every 172.2 kb across the whole genome and one SNP every 386 kb in the exons. Heterozygosity (Table S3) ranged from 18% to 3% among various accessions. Sangria, Jyothi, Hungarian semi hot, Tormenta hot, Costeno Amarillo, Cayenne Purple, PI439355, Watermelon, Numex R Naky, Jalapeno pepper, Prik ki nu and Red Rocket were the most highly heterozygous, from 18% to 9%. Mean heterozygosity was 6%, so the accessions in the study were generally homozygous at most of the polymorphic loci and hence suitable for GWAS analysis. The mean MAF was 1892 (range 2711 [Tepin Guatemala] to 1115 [Pimento Sweet Apple]).

### Population stratification and effect of capsaicin content and FW

**Figure 1:**
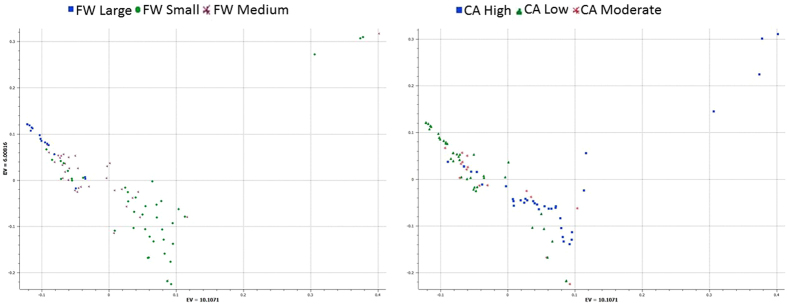
Principal component analysis (PCA) showing distribution of global *Capsicum annuum* accession collections with 7, 331 single nucleotide polymorphisms (SNPs). (**A**) PCA with accessions grouped by capsaicin content. (**B**) PCA with accessions grouped by fruit weight. See Table S2 for a list of accessions and for respective eigen values to locate individual accessions on the graph.

**Figure 2:**
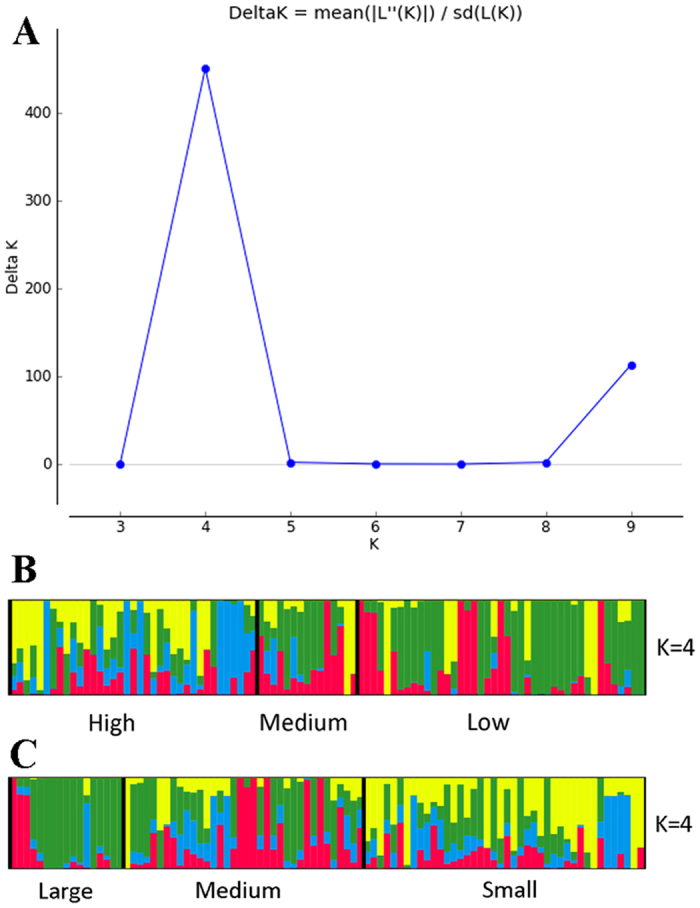
Admixture in subpopulations resolved using 7, 331 single nucleotide polymorphisms (SNPs) along with capsaicin content (high, medium and low) (B) and SNPs and Fruit weight (large, medium and small) among *C. annuum* accession collections by population structure, a model-based approach. (**A**) K4 had the highest peak (based on Delta K distribution), so four clusters sufficiently define *C. annuum* population structure based on capsaicin content and fruit weight.

We used PCA of the 7,331 SNPs to classify domesticated and wild *C. annuum* peppers. Two PCA figures based on capsaicin content (CA) and FW classifications were created to understand the relationships of accessions. This analysis produced a close cluster of hot peppers on CA-PCA and bell peppers as separate cluster (Fig. 1). Sangria, Bellingrath Gardens, Prik ki nu, Pepperoncini, Red Rocket, Hot Red Rocket, Jwala, PI 439355, Jyothi, Hinkelhatz pepper, PI 636424, De Arbol, Goat’s Weed, Costeno Amarillo, Cayenne Purple and Yellow Peter were clustered around the wild accessions. Similarly, three serrano peppers, namely Mexican serrano, Serrano pepper and Serrano Tampiqueno, were grouped together and distant from the rest of the accessions. PCA placement of the various accessions can be noted from the eigen values presented in Table S4. To validate the results of PCA, we used a model-based approach for population structure analysis to analyze the entire panel of 94 *C. annuum* accessions grouped by CA and FW (Fig. 2). Use of Structure Harvester provided Delta K values, which showed K-4 as the most appropriate.

**Figure 3:**
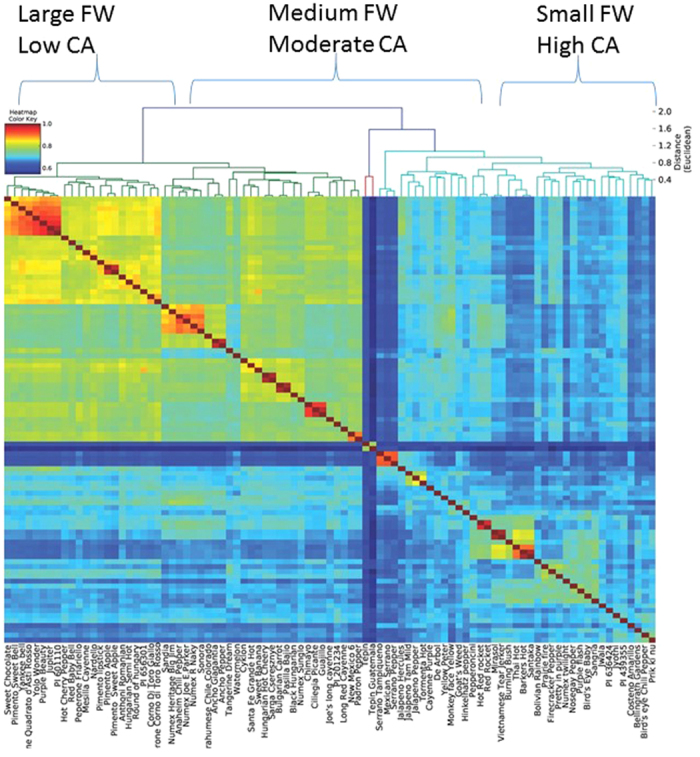
Individuals are ordered by their Euclidean distance (identity by state) and clustering with the tree is shown on top. Heat map key is presented on the top left corner (values rescaled from 0.6 to 1).

We examined allele sharing across the panel by calculating identity by state (IBS) coefficients among all pairs of accessions (Fig. 3). Allele sharing clearly tracks subpopulation ancestry as identified by PCA and Structure outputs. The mean observed IBS sharing was greatest for large-sized sweet peppers (0.97) as compared with small-sized hot peppers (0.85), with relatively little IBS sharing between high and low capsaicin-containing peppers (0.54 ± 0.94). The highest IBS sharing among high and low capsaicin content types was between Burning Bush and Chimayo (0.94) and the lowest between Tepin Guatemala and Black Hungarian (0.55). For FW, the mean IBS for small and large types was 0.70 ± 0.06. Most of the admixture occurred within small-sized hot peppers and large bell peppers indicating strong artificial selection for fruit size.

### Population differentiation and signals of positive selection

**Figure 4:**
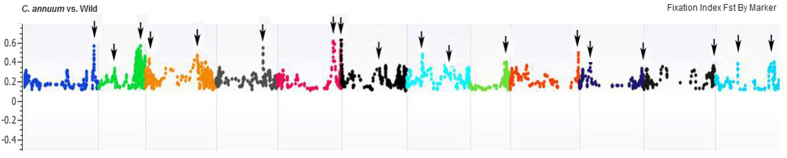
Genome-wide window-based pairwise *F*_*ST*_ values of low and high FW (blue) and overall FST values (red) across chromosomes. Note *F*_*ST*_ distribution on part of chromosome 11 showed distinct sweep.

**Figure 5:**
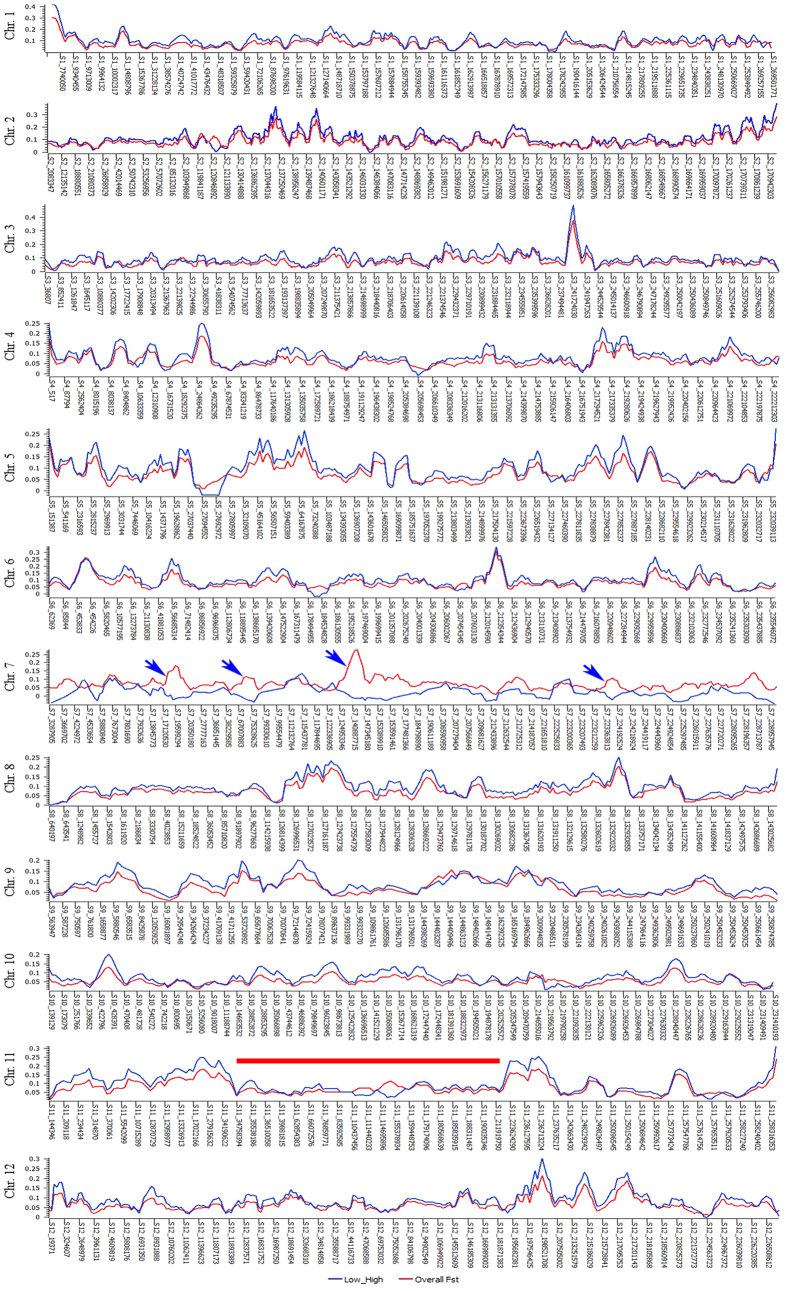
Genome-wide window-based pairwise FST values of small and large fruit types (blue) and overall FST values (red) across chromosomes. Note FST distribution on part of chromosome 11 showed distinct sweep.

The allele fixation index (*F*_*ST*_) between domesticated and wild accessions was 0.10. The *F*_*ST*_ between low and high, low and medium, and medium and high capsaicin types was 0.08, 0.02 and 0.03, respectively, and that between small and large, small and medium, and medium and large FW types was 0.15, 0.03 and 0.07, respectively. The highest *F*_*ST*_was noted for SNPs on chromosomes 1, 2, 4, 6, 7 and 8, so these regions are under positive selection. We annotated 122 regions showing strong positive selection and identified important genes for FW (Table S5). When comparing significant (P < 0.001) pairwise *F*_*ST*_ distribution of large and small FW accessions across the genome, we identified genomic areas with selective sweep signatures that are important for FW (Figs 4 and 5). A segment of 177 Mb was under strong selection sweep on chromosome 11. This sweep was from 34,758,394 to 211,919,750 Mb. In total, 659 genes located in this sweep area have important roles in transmembrane transport of lipids, intercellular carbohydrate transport, carbohydrate metabolic process and carbohydrate transport, ATP binding, flavonoid biosynthesis/auxin response, RNA splicing, nucleic acid binding protein, transcription regulation, glutathione metabolic process, positive regulation of GTPase activity, and other functions (Tables S6 and S7).

### Analysis of LD

**Figure 6:**
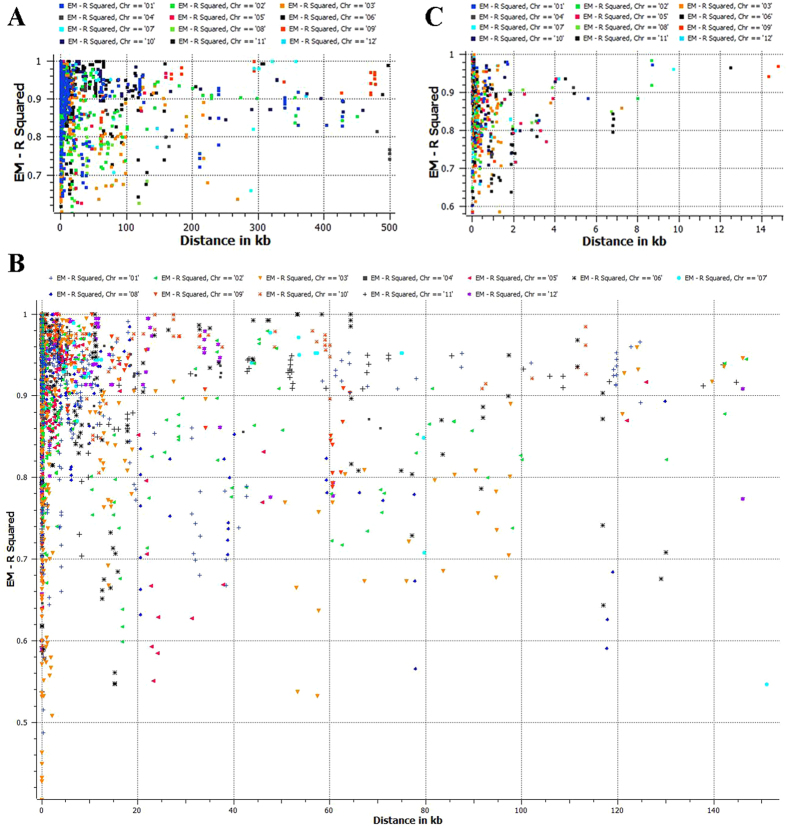
Marker associations (r^2^) across various chromosomes. EM analysis is carried for (**A**) adjacent individual SNPs, (**B**) adjacent haplotypes, and (**C**) SNPs located in individual genes.

**Figure 7:**
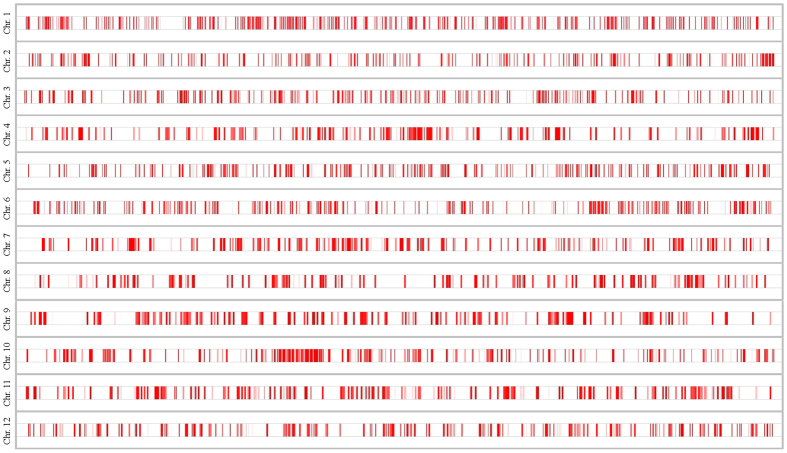
High and low linkage disequilibrium (LD) block regions interspersed across the 12 *C. annuum* chromosomes.

**Figure 8:**
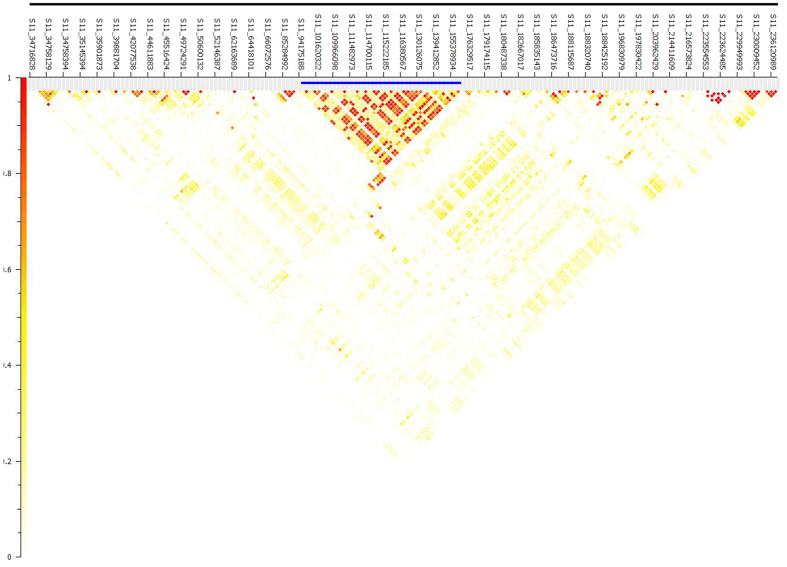
LD distribution across the 177 Mb sweep region specific to fruit weight identified on chromosome 11.

Haplotype distribution is important in comparing common and unique patterns of genetic variation of *C. annuum* gene pools and has a wide range of applications. The two major processes that shape haplotype structure are the domestication process and breeding history. We used “Minimize historical recombination”, a block-defining algorithm developed by Gabriel *et al*.^21^ to define haplotypes of various lengths. The upper confidence bound was set to 0.98 and the lower bound was set to 0.70. SNPs below MAF of 0.05 were skipped. Maximum block length was set to 160Kb. The EM (Expectation Maximization) algorithm was used for haplotype estimation with convergence tolerance 0.0001 and frequency threshold of 0.01. Maximum EM iterations were set to 50. The current study identified 1189 haplotypes containing 3413 SNPs with a range of 9 to 2 SNPs per haplotype (Table S8).We conducted an extensive LD analysis on the entire dataset of 94 *C. annuum* collections, on all adjacent marker pairs within a chromosome or within a haplotype block. The results provided values for both the expectation-maximization (EM) algorithm^22^ and composite haplotype method (CHM)^23^. R^2^ (squared-allele frequency correlations) and D’ (LD estimate) values for the EM and CHM methods are in Table S9. We created LD plots using marker-pair associations of adjacent SNPs within a chromosome, within a haplotype block, and within genes (Fig. 6A, B, and C). Length of individual LD blocks varied along chromosomes, with regions of high and low LD interspersed (Fig. 7). Pairwise LD was estimated by r^2^ and we compared the pattern of decay at different levels. First, with pair-wise analysis considering SNPs across chromosomes, we noted LD decay on average, with an average block size of 139 kb (Fig. 6A). Second, analysis based on adjacent SNPs within haplotypes revealed LD decay within 28 kb (Fig. 6B). We performed genomewide haplotype analysis and identified 1209 haplotypes (Table S10) Third, analysis of SNPs located in exons revealed mean LD decay within 1 kb (Fig. 6C). For LD analysis on chromosome 11, we noted that an entire region under sweep was also under high LD (Fig. 8). Genes under sweep and high LD included CA11g07400 (AP-1 complex subunit gamma-1 with the biological process of intracellular protein transport), with LD 10.2 kb, followed by CA11g09160 (ankyrin-like protein, an acyltransferase), with LD 4.9 kb, and CA11g09970 (flavonol synthase/flavanone 3-hydroxilase), with LD 3.3 kb (Table S7).

### GWAS to locate QTL for capsaicin and dihydrocapsaicin content

**Figure 9:**
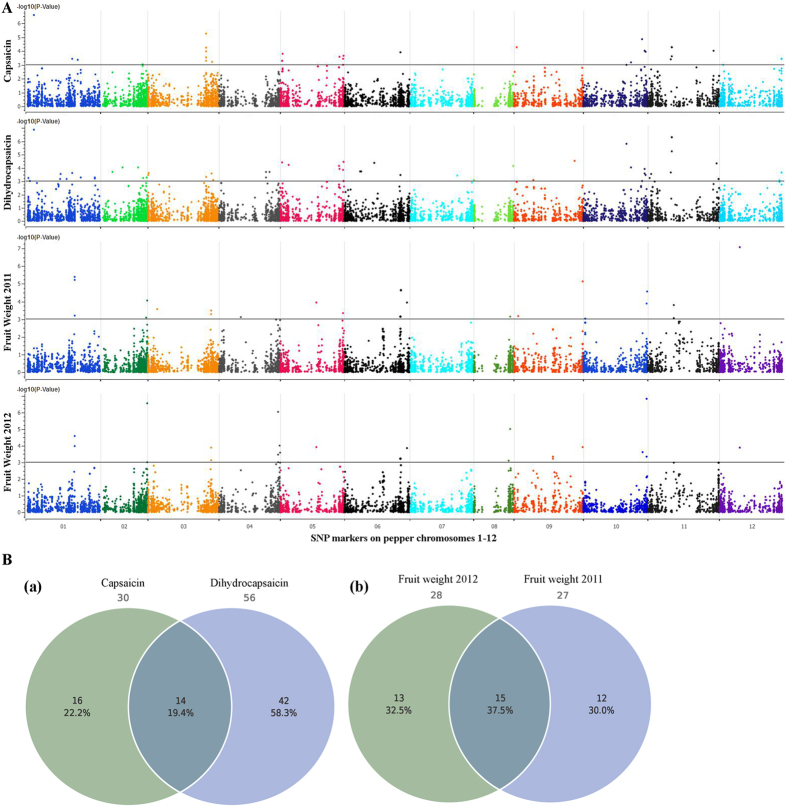
Manhattan plot of the genome-wide association study for fruit weight and capsaicinoids (capsaicin and dihydrocapsaicin). Chromosome coordinates are displayed along the X-axis, with the negative log-10 of the association P-value for each SNP on the Y-axis. Higher negative log-10 indicates stronger association with the trait. Venn diagrams are of the unique and common significantly associated SNPs for capsaicin and dihydrocapsaicin content and fruit weight in 2011 and 2012.

We used a GWAS with 7,331 SNPs to identify alleles that affect capsaicin and dihydrocapsaicin content (Fig. 7; individual SNP associations along with the details of major and minor allele frequencies and magnitude of associations are in Tables S10, S11, and S12; detailed annotations for all associated SNP markers are in Tables 1 and 2. We found 30 and 56 SNPs associated with capsaicin and dihydrocapsaicin content, respectively; 14 were common to both traits (Fig. 9). Average variation (%) explained per chromosome varied from 11.6 to 17.5 for capsaicin content and 12.9 to 16.9 for dihydrocapsaicin content.

Significantly associated SNPs for both capsaicin and dihydrocapsaicin content were S1_31111874 (-log10 P = CA 6.6 and DCA 6.9), S3_211558976 -log10 P = CA 4.0 and DCA 3.3), S5_215972421 (-log10 P = CA 3.6 and DCA 3.9), S5_227837981 (-log10 P = CA 3.4 and DCA 3.8), S5_229634509 (-log10 P = CA 3.6 and DCA 4.5), S6_203416571 (-log10 P = CA 3.9 and DCA 3.5), S10_156251204 (-log10 P = CA 3.0 and DCA 5.8), S10_172735351 (-log10 P = CA 3.1 and DCA 4.0), S10_221317647 (-log10 P = CA 4.0 and DCA 3.9), S10_225598553 (-log10 P = CA 3.9 and DCA 3.5), S11_83592400 (-log10 P = CA 3.4 and DCA 3.7), S11_85543247 (-log10 P = CA 4.2 and DCA 6.3), S11_85543251 (-log10 P = CA 3.6 and DCA 5.3), and S11_85543257 (-log10 P = CA 4.2 and DCA 6.3).

### GWAS to locate QTL for FW

Despite the high variation noted for FW across the 2 years of the study, 15 common SNPs were found strongly associated (Fig. 8, Table 2, S13 and S14). Our GWAS revealed 28 and 33 SNPs with strong association during 2011 and 2012, respectively. We found an association of S1_178148471 (-log10 P 2011 = 5.2 and 2012 = 4.0) located in sopenicillin N epimerase (AAT_I superfamily) with ADP binding catalytic activity, S1_178214095 (-log10 P 2011 = 5.4 and 2012 = 4.6) of the protein transport protein SEC23-like (zf, MIDAS domain) that regulates Zinc ion binding, S2_169874314 (-log10 P 2011 = 4.0 and 2012 = 6.5) located in the intergenic space of Na^+^/H^+^ antiporter and lucose-6-phosphate 1-dehydrogenase, S3_230322338 (-log10 P 2011 = 3.3 and 2012 = 3.9) of the intergenic space between SNF1-related protein kinase and RAD50-interacting protein S3_230372266 (-log10 P 2011 = 3.5 and 2012 = 3.1) located in Ubiquitin-like modifier-activating enzyme 5-like (thiamine synthesis) and S5_131824978 (-log10 P 2011 = 3.9 and 2012 = 3.9) of an unknown protein. We also found an association of S6_202147247 (-log10 P 2011 = 3.9 and 2012 = 3.9), S6_202147285 (-log10 P 2011 = 3.1 and 2012 = 3.2), S6_202147337 (-log10 P 2011 = 3.1 and 2012 = 3.2) and S6_202147420 (-log10 P 2011 = 3.1 and 2012 = 3.2) located in the intergenic space between STYLOSA protein and flavin monooxygenase in both years. We found an association of S6_227195619 (-log10 P 2011 = 3.9 and 2012 = 3.8) of chloroplastic-FANTASTIC FOUR (FAF)-like protein, S8_132459145 (-log10 P 2011 = 6.8 and 2012 = 3.7) of DnaQ-like exonuclease, S9_250224149 (-log10 P 2011 = 6.8 and 2012 = 3.7) of mitochondrial-processing peptidase subunit alpha, S10_229225552 (-log10 P 2011 = 3.1 and 2012 = 5.0) of cell division control protein 45 (CDC45), S11_94177155 (-log10 P 2011 = 3.8 and 2012 = 3.0) of Clathrin assembly protein, and S12_72971688 (-log10 P 2011 = 7.06 and 2012 = 3.9) in the intergenic space between CLAVATA1 receptor kinase and pentatricopeptide repeat. S6_227195619, S6_204246361, S10_229225552 were nonsynonymous SNPs located in isopenicillin N epimerase (AAT_I superfamily), chloroplastic-FAF-like protein, cell division control protein 45 (CDC45) and TRS120 isoform, respectively, which have biological processes involving cell division and meristem organization.

## Discussion

Understanding the genetic control of traits influenced by domestication is improving as a result of GWAS for several crops. Domesticated peppers have larger fruits than wild peppers but also have larger leaves, flowers and seeds. An overall increase in size of many different organs could result from an increase in cell number or size or both^24^. Principal component analysis (PCA), model based population structure and an Euclidean tree built based on identity by state (IBS) indices revealed that the clustering pattern of diverse accessions were in agreement with capsaicin content (CA) and FW classifications indicating the importance of these traits in shaping modern pepper genome. Our study focused on unraveling various mechanisms underlying fruit weight and capsaicin content among *C. annuum* peppers.

A total of 659 genes located in the sweep of chromosome 11 were identified that influenced FW in *C. annuum*. In particular, the pentatricopeptide repeat protein and ABC transporter are known genes for tomato FW^25^, and the ankyrin repeat protein, which functions as an acyltransferase, with a homologue on chromosome 5 highly associated with capsaicin content as well as FW are located in the sweep region. Similarly, recent tomato genome analysis revealed that most of the genes, namely glutathione S-transferase, actin-related protein 2/3 complex, endo-1, 4-beta-glucanase, RCC1 domain-containing protein, ankyrin repeat protein, and xanthine dehydrogenase, were also found in the chromosome sweep important for tomato domestication^26^. We identified 30 genes in the sweep region with high LD, and our GWAS revealed highly significant markers for FW within the sweep. Qin *et al*.^9^ identified 115 regions across the genome containing 511 genes that are important for domestication with strong selective sweep signals by using WGS analysis. Among the genes in the chromosome 11 sweep, ankyrin-like protein, an acyltransferase, showed extended LD, up to 10 kb, which implies its role in pepper fruit weight.

Despite a number of genes involved in the capsaicin synthesis pathway per se, these genes need upstream and *trans*-regulators for proper spatiotemporal expression during capsaicin synthesis in pepper fruit formation. Our GWAS results showed an association with multiple genes via tightly linked SNP markers. Among the DNA/RNA binding proteins, CCHC zinc-finger, CCCH zinc-finger, GRAS transcription factor, transparent testa 12, and pentatricopeptide repeat protein are the major markers. Because these genes are transporters, transcription factors and catalytic enzymes, they might be regulators of the genes in the capsaicin biosynthesis pathway. Another homologue of ankyrin-like protein on chromosome 5 was also strongly associated with capsaicinoid content. Han *et al*.^27^, Stewart *et al*.^28^ and Reddy *et al*.^29^ demonstrated that *Pun1* is responsible for capsaicinoid synthesis; the authors further suggested the presence of an unknown enzyme that reduces vanillin to vanillyl alcohol. *Pun1* located on chromosome 2 was noted to encode AT3, an acyl transferase from the BAHD acyl transferase superfamily. How AT3 is related to the acyltransferase on chromosome 11 and 5 needs further investigation. Because its molecular function is also as an acyltransferase, ankyrin repeat-containing protein might function as an additional *Pun*1 gene, which is yet to be determined, or it might have an accessory role in capsaicin synthesis. Our results demonstrating the influence of multiple chromosomal regions on capsaicinoid content may begin to explain the extensive diversity evident in pepper for capsaicinoid concentration in pungent genotypes.

We identified 16 SNPs strongly associated with FW in both years of the study. The *C. annuum* progenitor species bear fruit of much smaller size than do the cultivated counterparts. Important findings from our FW GWAS study reveal similar biological function in tomato and other plants (Lin *et al*.^38^). We found a strong association of Stylosa protein (S6_202147247 S6_202147285 S6_202147337 S6_202147420) with FW in both seasons of the study; FASCIATED (FAS), encoded by a member of the YABBY family regulating organ polarity, is thought to play an important role in plant growth and development^30^,^31^. FAS controls fruit shape in tomato^32^. Earlier, STYLOSA (STY) was found to regulate floral homeotic meristem and organ identity in Antirrhinum^33^. Interactions between STYLOSA and YABBY family proteins control Antirrhinum vegetative and reproductive development^34^. The interaction between STYLOSA (Ca06g14190) and YABBY might be critical for fruit size/shape and weight in pepper.

S6_227195619 causes a nonsynonymous mutation in chloroplastic FAF protein and we found it strongly linked with FW in both seasons of the study. Plastid genes are expressed at high levels in photosynthetically active chloroplasts, including in developing fruits. Chloroplastic-FAF protein regulates shoot meristem size in Arabidopsis. FAF genes are expressed in the center of the shoot meristem, overlapping with the site of WUSCHEL (WUS) expression. Strong interaction between FAF and WUS determine the fate of meristem activity^35^. Hence, FAF (Ca06g22610) might regulate the size of the shoot meristem as well as FW by modulating the CLV3-WUS feedback loop in pepper. A significantly associated SNP, S9_250224149, was found in the mitochondrial processing peptidase alpha subunit (Ca09g16860), which might be important for fruit development because its ortholog in tomato is specifically and differentially expressed during cell expansion stages in early stages of fruit development^36^.

S12_72971688 is an intergenic SNP close to CLAVATA 1 receptor kinase that showed a strong association with FW in both seasons. Arabidopsis plants homozygous for mutations at the CLAVATA1 (CLV1) locus, a receptor kinase protein, accumulate excess undifferentiated cells^37^,^38^. This gene is critical for shoot and flower meristem size. In our study, Ca12g10020, or CLAVATA1 receptor kinase, might interact with CLV3 as a ligand–receptor pair in a signal transduction pathway coordinating growth between adjacent meristematic regions and controlling the balance between meristem cell proliferation and differentiation.

Fine mapping of the QTL fw3.2 controlling FW in tomato was accomplished recently^25^ and genes in this QTL range were identified. Among seven putative genes identified in this region, ORF4 encodes a protein with high identity to PNM1 in Arabidopsis PNM1 belongs to the pentatricopeptide repeat containing protein (PPR) family that functions in RNA binding (Hammani *et al*. 2011). The marker S12_72971688 linked to the locus Ca12g10030 (PPR) might play key roles in determining FW in pepper as QTL fw3.2 does in tomato.

Given our SNP density and sample size, this study is not sufficiently robust to find alleles of small effect among the pepper secondary centers of origin distributed across the world. However, we located SNPs with major effect in highly inbred, diverse and smaller population. Some of the strongest signals are quite far from known capsaicin genes possibly because of ascertainment bias. Also SNPs located in the candidate genes may not be in LD, which hampers their identity in association mapping strategies. Furthermore, stringent control for population stratification and IBS may eliminate useful SNPs located in the candidate genes. Such tradeoffs are part of GWAS, especially in reducing spurious associations^39^. Zhao *et al*.^39^ and Rife *et al*.^40^ proposed that SNP identification from transcriptome datasets or spiked GBS, which combines targeted amplicon sequencing with reduced representation GBS, will improve our ability to detect moderate-strength and rare alleles, especially those located in candidate genes. Analogous approaches have been reported^39^,^41^,^42^,^43^.

Our GWAS for capsaicin and dihydrocapsaicin content and FW identified SNPs located in the candidate genes known to have similar biological functions in tomato and other plants for these domestication related traits. Accessions containing higher minor allele frequencies from this study can be used to generate nested association mapping populations to validate GWAS results and further dissect the complex interaction among genes involved in the pleotropic effects on fruit size and capsaicin content evident in *Capsicum*.

## Materials and Methods

We included 94 accessions of *C. annuum* belonging to various countries representing a wide geographical area of the world for the molecular diversity analysis (Table S1). These selfed accessions were grown in three replications during two seasons (2011 and 2012) adapting a row-to-plant spacing of 100 × 30 cm. Ten plants per accession were grown in each replication. FW (g), was collected for five plants. Quantitative analysis of capsaicin and dihydrocapsaicin content was performed using greenhouse-grown plants in three replications using the 1200 series HPLC system (Agilent Technologies, Santa Clara, CA) with a degasser, an autosampler, and a binary pump as described^7^.

### Genotyping by sequencing

Genomic DNA was isolated using the DNeasy plant mini kit (QIAGEN, Germany), and GBS was as described^44^,^45^. Briefly, genome complexity was reduced by digesting total genomic DNA from individual samples with the ApeKI, a type II restriction endonuclease that recognizes a degenerate 5-bp sequence (GCWGC, where W is A or T), which creates a 5′ overhang (3 bp) and is partially methylation-sensitive (will not cut if the 3′ base of the recognition sequence on both strands is 5-methylcytosine). Digested products were then ligated to adapter pairs with enzyme-compatible overhangs; one adapter contained the barcode sequence and a binding-site Illumina sequencing primer (Illumina Inc., USA). These samples were pooled, purified and amplified with primers compatible with the adapter sequences. Temperature cycling was 72 °C for 5 min, 98 °C for 30 s followed by 18 cycles of 98 °C for 30 s, 65 °C for 30 s, and 72 °C for 30 s with a final Taq extension step at 72 °C for 5 min. Amplified sample pools constituted a sequencing “library”. Libraries were purified and 1 μL was loaded onto an Experion automated electrophoresis station (BioRad, Hercules, CA) for evaluation of fragment sizes. Libraries were considered suitable for sequencing if adapter dimers (~128 bp in length) were minimal or absent and most of the other DNA fragments were between 170 and 350 bp. If adapter dimers were present in excess of 0.5% (based on the Experion output), libraries were constructed again by using a few DNA samples and decreasing adapter amounts. The PCR primers also added 3′ sequences complementary to the solid-phase oligonucleotides that coat the Illumina sequencing flow-cell. After PCR, pooled products were purified; GBS “library” fragment size distributions were checked on a BioAnalyzer (Agilent Technologies, USA). Products were quantified and diluted for sequencing by use of Illumina HiSeq 2500. A bioinformatics pipeline, TASSEL-GBS, designed for efficient processing of raw GBS sequence data into a SNP genotype file^46^ was used. Barcoded sequence reads were processed and collapsed into a set of unique sequence tags, with one TagCounts file produced per input FASTQ. Chromosomal assignment physical map position of candidate genes and GBS markers, were deduced from the hot pepper WGS draft at http://peppergenome.snu.ac.kr. SNPs were designated on the basis of chromosome number and position (e.g., S10_172735351 indicates SNP located at 172735351 position on chromosome 10).

## Data Analysis

### Population structure analysis

For quantitative assessment of the number of clusters in the GWAS panel, we used a Bayesian clustering analysis with a model-based approach implemented in STRUCTURE v2.2^47^. This approach involves use of multi-locus genotypic data to assign individuals to clusters or groups (K) without prior knowledge of their population affinities. The program was run with SNP markers for k-values 1–9 (hypothetical number of subgroups), with 100,000 burn-in iterations, followed by 500,000 Markov Chain Monte Carlo (MCMC) iterations for accurate parameter estimates with a high-performance cluster. To verify the consistency of the results, we performed three independent runs for each K. An admixture model with correlated allele frequencies was used. The optimal K value was determined by use of an ad-hoc statistic, ΔK. The number of *K*s in each dataset was evaluated by ΔK values estimated and visualized with the software Structure Harvester, (www.taylor0.biology.ucla.edu/structureHarvester)^48^. To facilitate the interpretation of population-genetic clustering results, we used CLUMPP (CLUster Matching and Permutation Program)^49^, which groups individuals into populations on the basis of multilocus genotypes, and the output was directly input into a program for cluster visualization DISTRUCT 1.1^50^. In a second approach, we used principle component analysis (PCA) with the SNP & Variation Suite (SVS v8.1.5) (Golden Helix, Inc., Bozeman, MT, USA; www.goldenhelix.com).

### Analysis of population differentiation

Fixation index (*F*_*ST*_) estimation was based on Wright’s F statistic^51^ in SVS v8.1.5 (http://goldenhelix.com/). Annotation and gene ontology terms for genes from the selective sweeps were identified with the WGS draft at http://peppergenome.snu.ac.kr.

### GWAS mapping

For GBS data, we considered only SNPs successfully mapped to the *Capsicum* WGS draft, because knowing the chromosome location of SNPs helps prevent spurious LD and thereby unreliable association mapping. Before studying LD decay, haplotype blocks were calculated for all markers using the default settings in SVS v8.1.5. Adjacent and pairwise measurements of LD for GBS data were calculated separately for SNPs in each chromosome. All LD plots and LD measurements and haplotype frequency calculations involved use of SVS v8.1.5 and Tassel 5.0. For GWAS, the population structure Q matrix was replaced by the PC matrix^52^. The PC matrix and identity by descent (IBD) was calculated from LD-pruned SNPs in SVS v8.1.5. GWAS involved a single-locus mixed linear model developed by the EMMAX method^53^ and implemented in SVS v8.1.5. We used a PC matrix (first two vectors) to correct for population stratification and the IBD matrix to correct polygenic background. Manhattan plots for associated SNPs were visualized in GenomeBrowse v1.0 (Golden Helix, Inc). The SNP P-values from GWAS underwent sequential Bonferroni correction^54^ as well as false discovery rate (FDR) analysis^55^. Annotation and gene ontology terms for the SNP containing sequences were identified with the WGS draft at http://peppergenome.snu.ac.kr.

## Additional Information

**How to cite this article**: Nimmakayala, P. *et al*. Genome-wide diversity and association mapping for capsaicinoids and fruit weight in *Capsicum annuum* L. *Sci. Rep.* **6**, 38081; doi: 10.1038/srep38081 (2016).

**Publisher's note:** Springer Nature remains neutral with regard to jurisdictional claims in published maps and institutional affiliations.

## Supplementary Material

Supplementary Information

Suplimentary Tables

## Figures and Tables

**Table 1:** Annotation of significantly associated SNPs for capsaicin and dihydrocapsaicin content.

Trait/markers	Locus^1^	Gene annotation	Location of SNP	Ma→Mi	Amino acid ^#^	Molecular function	Biological process
**Capsaicin and dihydrocapsaicin**							
S1_31111874	CA01g09570	F-box/FBD/LRR-repeat protein	Promoter	C→G	—	Unknown function	Regulation of axonogenesis
S3_211558976	CA03g19170	Mitochondrial carrier protein MTM1-like	Exon 1	A→G	V→I^#^	Metallochaperone activity	Manganese and phosphate ion transport
S5_215972421	CA05g15510	Laccase cupredoxin	Exon 5	C→G	T→S^#^	Ferroxidase activity	Seed germination and root elongation
S5_227837981	CA05g18080	Ankyrin repeat-containing protein	Exon 1	A→G	D→G^#^	Acyltransferase, Transferase	Cell growth regulation
S5_229634509	CA05g18740	IKI3 family protein	Exon 3	A→T	K→R^#^	Histone acetyltransferase, tRNA binding	Transcription and protein transport
S6_203416571	CA06g14430	ABC transporter G family	Exon 20	C→T	A→V^#^	ATPase activity, coupled to transmembrane movement	Transmembrane transport of lipids
S10_156251204	CA10g10170/CA10g10180	Zinc-finger CCHC type/GRAS transcription factor	Intergenic	G→A	—	Nucleic acid binding/DNA binding	mRNA processing/Regulation of transcription
S10_172735351	CA10g10850	Nuclear transport factor 2 (NTF2) family protein	Exon 1	T→C	N→N	Protein transport	Nucleo-cytoplasm transport
S10_221317647	CA10g16820	4-hydroxy-tetrahydrodipicolinate synthase	Intron 4	A→C	—	4-hydroxy-tetrahydrodipicolinate synthase	Lysine biosynthesis, diaminopimelate bisynthesis
S10_225598553	CA10g18180	Short-chain type alcohol dehydrogenase	3-UTR	C→T	—	Oxidoreductase	—
S11_83592400	CA11g09150	ABC transporter C family member 3-like	Intron 1	A→T	—	ATPase activity, coupled to transmembrane movement	Transmembrane transport of lipids
S11_85543247	CA11g09200/CA11g09210	Transparent Testa 12/F-box protein	Intergenic	C→T	—	DNA binding transcription factor/Protein binding	Proanthocyanidin biosynthesis, seed development/-
S11_85543251	CA11g09200/CA11g09210	Transparent Testa 12/F-box protein	Intergenic	T→C	—	DNA binding transcription factor/Protein binding	Proanthocyanidin biosynthesis, seed development/-
S11_85543257	CA11g09200/CA11g09210	Transparent Testa 12/F-box protein	Intergenic	A→G	—	DNA binding transcription factor/Protein binding	Proanthocyanidin biosynthesis, seed development/-
**Capsaicin only**							
S3_212689068	CA03g19340	Putative hydrolase	Promoter	T→C	—	Hydrolase activity	Regulation of cell proliferation
S10_213596026	CA10g15030	Syntaxin-71-like (t-SNARE family)	Intron 3	A→T	—	Protein transporter, SNAP receptor	Vesicle transport, protein target to membrane
**Dihydrocapsaicin only**							
S2_78953537	CA02g05900/CA02g05910	ATP-dependent DNA helicase pcrA/Peroxidase	Intergenic	A→C	—	ATP binding, DNA binding/metal ion binding, peroxidase activity	DNA replication/lignin biosynthesis, H_2_O_2_ catabolism
S2_136027877	CA02g11920	Detected protein of unknown function	Exon 8	C→G	H→D^#^	—	—
S5_6535912	CA05g02820	Detected protein of unknown function	Promoter	C→T	—	—	—
S5_30787082	CA05g06630/CA05g06640	Pentatricopeptide repeat protein/CCCH-type zinc finger protein	Intergenic	T→C	—	RNA binding/DNA binding	Transit peptide processing/Negative regulation of transcription
S5_213819539	CA05g15030/CA05g15040	Intracellular transport USO-1 protein/Basic 7 S globulin 2	Intergenic	C→G	—	Protein transporter activity/aspartic-type endopeptidase activity	SNARE complex and vesicles docking/-
S6_107738639	CA06g07610/CA06g07620	F-Box protein/Elongation factor 1-alpha	Intergenic	C→G	—	Glycoprotein binding/translation elongation, GTPase activity	DNA repair, protein ubiquination/Protein biosynthesis
S8_142512369	CA08g18030/CA08g18040	Serine/threonine-protein kinase SMG1/Serine/threonine-protein kinase SMG1-like	Intergenic	G→A	—	ATP, metal ion binding/ATP, metal ion binding	mRNA transport, response to stress/mRNA transport, response to stress
S9_220486511	CA09g12880	Os09g0132600 protein	Exon 4	C→G	H→D^#^	Uncharacterized protein	—
S11_249688869	CA11g16730/CA11g16740	Nectarin 1/Disease resistant protein BS2	Intergenic	A→G	—	Manganese ion binding, SOD activity/Nucleotide binding	Nutrient reservoir activity/disease resistance

^1^Korea genome locus number; + or −, direction of transcription on + or – strand; Ma→Mi - Major and minor alleles from mapping population; ^#^nonsynonymous mutation.

**Table 2:** Annotation of significantly associated SNPs for fruit weight.

Trait/markers	Locus^1^	Gene annotation	Location of SNP	Ma→Mi	Amino acid ^#^	Molecular function	Biological process
**Fruit weight (2011 and 2012)**							
S1_178148471	CA01g23190	Isopenicillin N epimerase (AAT_I superfamily)	Exon 1	C→T	S→N^#^	ADP binding, catalytic activity	Penicillin biosynthesis
S1_178214095	CA01g23200	protein transport protein SEC23-like (zf, MIDAS domain)	Promoter	A→G	—	Zinc ion binding	Intracellular protein transport
S2_169874314	CA02g30530/CA02g30540	Na^+^/H^+^ antiporter/Glucose-6-phosphate 1-dehydrogenase	Intergenic	C→T	—	Sodium:proton antiporter activity/NADP binding	Multiple cellular processes/glucose metabolic process
S3_230322338	CA03g24610	SNF1-related protein kinase/RAD50-interacting protein	Intron 4	C→T	—	ATP binding, protein kinase/membrane traffic between the Golgi and ER	Nitrate assimilation, phosphorylation, carbohydrate metabolism/protein transport and Golgi transport
S3_230372266	CA03g24640	Ubiquitin-like modifier-activating enzyme 5-like (thiamine synthesis)	Intron 10	A→T	—	Cofactor binding	Small protein activating enzyme activity
S5_131824978	CA05g10770	Unknown protein	Promoter	C→T	—	—	—
S6_202147247	CA06g14190/CA06g14200	STYLOSA protein/Flavin monooxygenase	Intergenic	C→T	—	Auxin transporter inhibitor/NADP binding	Regulation of floral organ identity/glucosinolate biosynthesis
S6_202147285	CA06g14190/CA06g14200	STYLOSA protein/Flavin monooxygenase	Intergenic	G→A		Auxin transporter inhibitor/NADP binding	Regulation of floral organ identity/glucosinolate biosynthesis
S6_202147337	CA06g14190/CA06g14200	STYLOSA protein/Flavin monooxygenase	Intergenic	C→→T		Auxin transporter inhibitor/NADP binding	Regulation of floral organ identity/glucosinolate biosynthesis
S6_202147420	CA06g14190/CA06g14200	STYLOSA protein/Flavin monooxygenase	Intergenic	C→T		Auxin transporter inhibitor/NADP binding	Regulation of floral organ identity/glucosinolate biosynthesis
S6_227195619	CA06g22610	Chloroplastic- FAF-like protein	Exon 1	C→G	N→K^#^	—	—
S8_132459145	CA08g12350	DnaQ-like exonuclease	Intron 2	C→T	—	DNA binding	Exonuclease activity
S9_250224149	CA09g16860	Mitochondrial-processing peptidase subunit alpha	Intron 11	C→T	—	Metal ion binding	Processing proteins targeting to mitochondrion
S10_229225552	CA10g19740	Cell division control protein 45 (CDC45)	Exon 1	A→G	S→S	Chromatin binding, helicase activity	Cell division, DNA replication
S11_94177155	CA11g09530	Clathrin assembly protein	Promoter	A→C		low-density lipoprotein particle receptor binding	Intracellular protein transport, endocytosis
S12_72971688	CA12g10020/CA12g10030	CLAVATA1 receptor kinase/Pentatricopeptide repeat	Intergenic	G→T		Protein kinase, ATP binding/RNA binding	Regulation of meristem structural organization, cell differentiation/Transit peptide processing
**Fruit weight 2011 only**							
S6_204245052	CA06g14620	TRS120 isoform	Intron	G→A		Cell plate assembly	Cell division
S6_204246361	CA06g14620	TRS120 isoform	Exon 6	G→A	R→G^#^	Cell plate assembly	Cell division
S10_231176974	CA10g20890/CA10g20900	Unknown function/Transcription factor	Intergenic	T→C		-/DNA binding	-/Transcription
**Fruit weight 2012 only**							
S4_215751345	CA04g19880/CA04g19890	LIM domain containing protein/DNA damage-binding protein	Intergenic	A→G		DNA and zinc ion binding/damaged DNA binding	Regulation of transcription/protein ubiquitination, nucleotide excision repair

^1^Korea genome locus number; + or −, direction of transcription on + or − strand; Ma→Mi - Major and minor alleles from mapping population; ^#^nonsynonymous mutation.
