# Supplementary material for: Genome-wide Diversity and Association Mapping for Capsaicinoids and Fruit Weight in Capsicum annuum L
Source: Sci Rep. 2016 Nov 30;6:38081. doi: 10.1038/srep38081 (PMC5128918; doi:10.1038/srep38081)
Supplement: Supplementary Information [file srep38081-s1.doc]

**Supplementary Information**

**Genome-wide Diversity and Association Mapping for Capsaicinoids and Fruit Weight in *Capsicum annuum* L.**

Padma Nimmakayala^1†^, Venkata L. Abburi^1†^, Thangasamy Saminathan^1^, Suresh B. Alaparthi^1^, Aldo Almeida^1^, Brittany Davenport^1^, Marjan Nadimi^1^, Joshua Davidson^1^, Krittika Tonapi^1^, Lav Yadav^1^, Sridhar Malkaram^1^, Gopinath Vajja^1^, Gerald Hankins^1^, Robert Harris^1^, Minkyu Park^2^, Doil Choi^2^, John Stommel^3^, Umesh K. Reddy^1†*^

^1^Gus R. Douglass Institute and Department of Biology, West Virginia State University, Institute, WV-25112, USA

^2^Department of Plant Science, Plant Genomics and Breeding Institute, College of Agriculture and Life Sciences, Seoul National University, Seoul 151-321, Republic of Korea

^3^Genetic Improvement of Fruits and Vegetables Laboratory (USDA, ARS), Beltsville, MD-20705

^†^ Authors contributed equally

* Corresponding author: [ureddy@wvstateu.edu](mailto:ureddy@wvstateu.edu)

**Supplementary information**

**Supplementary tables**

Table S1: List of accessions, origin and means of various traits used in the current study.

Table S2: Number of sequence reads generated per accession in the current study.

Table S3: Extent of heterozygosity, minor allele frequencies in various accessions.

Table S4: Eigen values for various principle components estimated with 7,331 SNPs for various accessions.

Table S5: Annotation and biological function of genes containing SNPs with higher *F_ST_* indices

Table S6: LD analysis for adjacent SNP pairs within the sweep located in chromosome 11.

Table S7: List of genes under high linkage disequilibrium (LD) and located in the sweep region of chromosome 11

Table S8: Haplotype blocks across the genome.

Table S9: LD analysis for adjacent SNP pairs across the genome.

Table S10: GWAS for capsaicin content showing significant SNPs and strength of association.

Table S11: GWAS of dihydrocapsaicin showing significant SNPs and strength of association.

Table S12: Common associated SNPs for capsaicin and dihydrocapsaicin content.

Table S13: GWAS for fruit weight (2011) showing significant SNPs and strength of association.

Table S14: GWAS for fruit weight (2012) showing significant SNPs and strength of association.
